# A Rare Cause of Right Upper Quadrant Pain in a 17-Year-Old Female

**DOI:** 10.1155/2013/597196

**Published:** 2013-02-28

**Authors:** Nwabundo Nwankwo, Aram Barbaryan, Alaa M. Ali, Aibek E. Mirrakhimov

**Affiliations:** Department of Internal Medicine, Saint Joseph Hospital, 2900 North Lake Shore Drive, Chicago, IL 60657, USA

## Abstract

A 17-year-old Hispanic female presented to our hospital with complaints of right upper quadrant abdominal pain, vomiting, and fever. Physical exam was positive for hepatomegaly. Abdominal computed tomography showed multiple hypoechoic liver masses. Liver biopsy was done, which was diagnostic for hepatic epithelioid hemangioendothelioma.

## 1. Introduction

Hepatic epitheliod hemangioendothelioma (HEH) is an extremely rare malignancy originating from vascular endothelial cells [1]. HEH was first reported by Ishak et al. in 1984, who reported 32 cases of HEH [2]. Unfortunately, HEH lacks specific clinical signs and laboratory markers. The definitive diagnosis is based on the liver biopsy. 

Below, we present a case of a 17-year-old female who presented to the hospital with complaints of right upper quadrant abdominal pain. Abdominal imaging revealed multiple liver masses, and biopsy was diagnostic for HEH. We will briefly discuss the available literature on HEH.

## 2. Case Presentation

A 17-year-old Hispanic female presented to the emergency department with complaints of the right upper quadrant abdominal pain. The pain had worsened over the past three days. Initially the pain was intermittent and later progressed to be constant. Review of symptoms was positive for vomiting and fever. No change in bowel habits or urinary pattern was reported.

**Figure 1:**
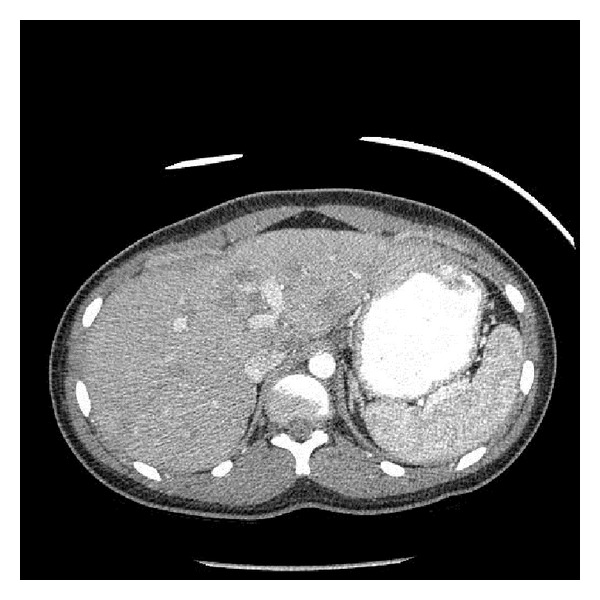
Abdominal CT showing multiple poorly defined low attenuation lesions.

**Figure 2:**
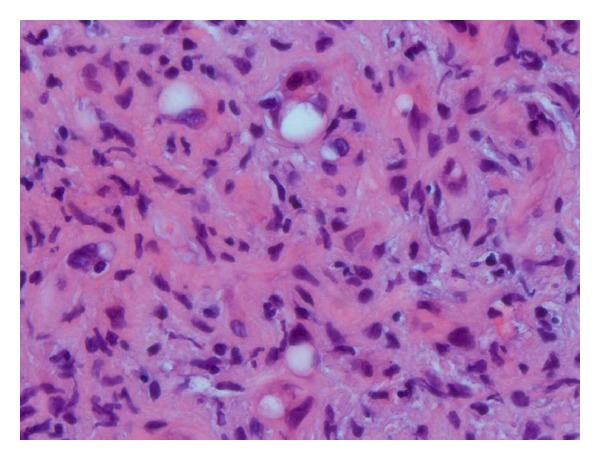
Liver biopsy showing scattered epitheliod cells. The tumor cells were stained positive for CD 31.

On physical examination, the patient had right lower and upper quadrant tenderness with hepatomegaly two centimeters below the right costal margin. Laboratory tests including complete blood count, comprehensive metabolic panel, lipid panel, and pancreatic enzymes were within normal limits. Abdominal ultrasound was done, which showed multiple hypoechoic masses scattered throughout the liver, measuring up to 2.0 cm in size. Computed tomography (CT) scan showed multiple low attenuation areas in the liver (Figure 1). Liver biopsy was performed, which was consistent with the diagnosis of HEH staining positive for CD31, a known endothelial marker (Figure 2). Skeletal survey and chest CT did not show evidence of metastatic disease.

Given the multifocal hepatic disease in this patient, she was not deemed to be a candidate for liver resection. The patient was referred for liver transplantation and is currently on the waiting list.

## 3. Discussion

HEH is a very rare liver tumor originating from vascular endothelial cells [1]. The disease pathogenesis is poorly understood. However, an overexpressed vascular endothelial growth factor (VEGF) may be a culprit in HEH [3].

Mean age at presentation is 41.7 years according to a review of 434 cases [4]. Right upper quadrant abdominal pain, weight loss, and liver enlargement are the most common clinical signs encountered in patients with HEH [4]. Lungs, peritoneal cavity, abdominal lymph nodes, and bones were the most commonly reported metastatic sites for HEH [4]. On extremely rare occasions, the tumor may metastasize to the neck [5] and may be clinically presented in adults as Kasabach-Merritt syndrome [6], which is a vascular tumor-related thrombocytopenia and bleeding.

Unfortunately, no laboratory biomarker is available for screening. However, cancer biomarker CA19-9, if abnormal, may indicate a poor prognosis in patients with HEH [7]. Abdominal imaging modalities such as CT, Magnetic Resonance Imaging (MRI), and positron emission tomography (PET) may be used in the workup of patients with suspected HEH [8, 9]. Nevertheless, the definitive diagnosis of HEH requires liver biopsy [1]. Histologically the tumor cells are positive for vascular markers such CD 31, CD 34, and coagulation factor VIII [1].

HEH has a highly variable prognosis ranging from a benign course to rapidly progressive with a poor outcome. Treatment is based on the radiological features of HEH: in a limited disease (less than three liver segments involved) tumor resection can be achieved, whereas liver transplantation is recommended for patient with multifocal liver disease [1]. Transcatheter arterial embolization of the tumor may be considered in selected cases such as in patients awaiting liver transplantation with multifocal liver involvement [4].

Several case studies reported that chemotherapy with radiation therapy may be useful in patient with HEH [4]. Two recent case studies reported that thalidomide used in patients with HEH led to a clinically and radiologically stable disease after 7 and 9 years of followup, respectively, [10, 11]. In the most recent case report on the treatment of HEH, Sangro et al. reported beneficial effects of VEGF inhibitor drug sorafenib [12]. These reports further support the notion that abnormal and enhanced angiogenesis is likely playing a leading role in the disease pathogenesis.

In conclusion, HEH is a very uncommon disease with nonspecific clinical features. Imaging studies are of some utility, and hepatic biopsy is essential to establish a diagnosis. Given its extremely rare incidence, it is almost impossible to run randomized controlled trials, and the clinicians guide the management based on the published case series.

## References

[1] Bioulac-Sage P, Laumonier H, Laurent C, Blanc JF, Balabaud C (2008). Benign and malignant vascular tumors of the liver in adults. *Seminars in Liver Disease*.

[2] Ishak KG, Sesterhenn IA, Goodman MZD, Rabin L, Stromeyer FW (1984). Epithelioid hemangioendothelioma of the liver: a clinicopathologic and follow-up study of 32 cases. *Human Pathology*.

[3] Emamaullee JA, Edgar R, Toso C (2010). Vascular endothelial growth factor expression in hepatic epithelioid hemangioendothelioma: implications for treatment and surgical management. *Liver Transplantation*.

[4] Mehrabi A, Kashfi A, Fonouni H (2006). Primary malignant hepatic epithelioid hemangioendothelioma: a comprehensive review of the literature with emphasis on the surgical therapy. *Cancer*.

[5] Kim SJ, Kim YC (2011). Unusual extrahepatic metastasis to the soft tissue of the left cervical neck area from hepatic epithelioid hemangioendothelioma. *Hepatology*.

[6] Ozturk B, Coskun U, Yaman E (2009). Adult hepatic epitheloid haemangioendothelioma presenting with Kasabach-Merrit syndrome: a case report. *Journal of Clinical Pathology*.

[7] Wang LR, Zhou JM, Zhao YM (2012). Clinical experience with primary hepatic epithelioid hemangioendothelioma: retrospective study of 33 patients. *World Journal of Surgery*.

[8] Lin J, Ji Y (2010). CT and MRI diagnosis of hepatic epithelioid hemangioendothelioma. *Hepatobiliary and Pancreatic Diseases International*.

[9] Dong A, Dong H, Wang Y, Gong J, Lu J, Zuo C (2013). MRI and FDG PET/CT findings of hepatic epithelioid hemangioendothelioma. *Clinical Nuclear Medicine*.

[10] Raphael C, Hudson E, Williams L, Lester JF, Savage PM (2010). Successful treatment of metastatic hepatic epithelioid hemangioendothelioma with thalidomide: a case report. *Journal of Medical Case Reports*.

[11] Salech F, Valderrama S, Nervi B (2011). Thalidomide for the treatment of metastatic hepatic epithelioid hemangioendothelioma: a case report with a long term follow-up. *Annals of Hepatology*.

[12] Sangro B, Iñarrairaegui M, Fernández-Ros N (2012). Malignant epithelioid hemangioendothelioma of the liver successfully treated with Sorafenib. *Rare Tumors*.

